# Interest of major serum protein removal for Surface-Enhanced Laser Desorption/Ionization – Time Of Flight (SELDI-TOF) proteomic blood profiling

**DOI:** 10.1186/1477-5956-4-20

**Published:** 2006-10-05

**Authors:** Stéphane Roche, Laurent Tiers, Monique Provansal, Marie-Thérèse Piva, Sylvain Lehmann

**Affiliations:** 1 Institut de Génétique Humaine du CNRS, 141 rue de la Cardonille, 34396 Montpellier, France; 2 CHU Montpellier, Laboratoire de Biochimie, Hôpital St. Eloi, 80, av A. Fliche, 34295 Montpellier Cedex 5, France; 3 Faculté de Médecine Montpellier – Nîmes, UM1 – UFR Médecine, 2, rue École de Médecine, 34060 Montpellier, France

## Abstract

**Background:**

Surface-Enhanced Laser Desorption/Ionization – Time Of Flight (SELDI-TOF) has been proposed as new approach for blood biomarker discovery. However, results obtained so far have been often disappointing as this technique still has difficulties to detect low-abundant plasma and serum proteins.

**Results:**

We used a serum depletion scheme using chicken antibodies against various abundant proteins to realized a pre-fractionation of serum prior to SELDI-TOF profiling. Depletion of major serum proteins by immunocapture was confirmed by 1D and 2D gel electrophoresis. SELDI-TOF analysis of bound and unbound (depleted) serum fractions revealed that this approach allows the detection of new low abundant protein peaks with satisfactory reproducibility.

**Conclusion:**

The combination of immunocapture and SELDI-TOF analysis opens new avenues into proteomic profiling for the discovery of blood biomarkers.

## Background

Human serum and plasma have an important clinical value for identification and detection of biomarkers. However, the analysis of these biological fluids is analytically challenging due to the high dynamic concentration range (over 10 orders of magnitude) of constituent protein/peptide species [1]. In addition, the few most abundant blood proteins constitute 95% of the bulk mass of proteins but they represent less than 0.1% of the total number of proteins. These high abundant proteins, and in particular albumin, produce large signals in most proteomics approaches and they mask or interfere with the detection of the other low amount protein components. This situation explains why the discovery of new proteins or peptides biomarkers in blood is challenging. To minimize these problems, proteomics techniques are constantly improving to provide a wider range and an optimized detection of low concentration candidates [2,3]. Many methods rely on a multidimensional separation scheme combining for example multidimensional chromatography or electrophoresis and mass spectrometry (MS) [4,5]. This is the case of the Surface-Enhanced Laser Desorption/Ionization – Time Of Flight (SELDI-TOF) method [6,7] that relies on MS to detect proteins and peptides initially selected by binding to various chromatographic surfaces (anionic, cationic, IMAC, hydrophobic). SELDI-TOF therefore focuses on a particular subset of the proteome for each of the capture conditions. However, results obtained so far with this technology have been often disappointing and controversial [8,9]. In fact this technology still has difficulties to detect low-abundant plasma and serum proteins and could benefit from additional pre-fractionation methods of blood (for review see Issaq et al, [10]). Thus, liquid chromatography [11], binding to solid-phase libraries [12,13] or enrichment of low molecular weight proteins [14] have been shown to improve SELDI-TOF analysis with however some drawback in terms of practicability, reproducibility, cost or difficulties to adapt to a high throughput approach.

## Results and discussion

**Figure 1:**
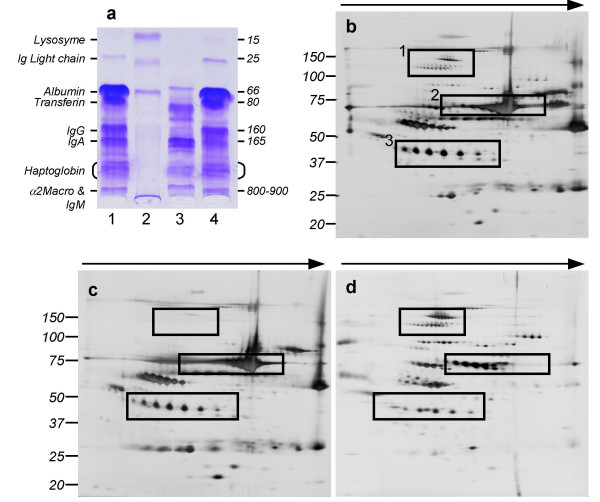
**Fractionation of serum protein by IgY12 spin columns followed by 1D and 2D electrophoresis**. Panel a: 1D electrophoresis is performed using Hydragel separation kit (Sebia) which allows the detection of major serum proteins including Ig light chains, albumin, transferin, IgG, IgA, Haptoglobin, IgM and alpha-2-macroglobulin. Lane 1: original unfractionated serum; lane 2: molecular weight standard; lane 3: unbound protein fraction (see Data Supplements) and lane 4: IgY12 bound protein fraction. The same amount of total protein was loaded in each lane. Panel b-d: 2D electrophoresis gel of original unfractionated serum (panel b), IgY12 bound proteins (panel c), and Y12 unbound proteins (panel d). Each gel was load with 20 μg of proteins. Some protein spots (box 1 panel b), were present in both the original and the unbound fractions (panel d). Others (box 2, panel a-b) (albumin, haptoglobin.), where retained by the column and recovered in the bound fraction (panel c). Finally, unmasked by the fractionation, some "new" spots were detected only in the unbound fraction (box 3, figure b-d). These results were consistent with the work of Huang et al [18].

Here, we evaluated the interest of the removal of major serum protein for SELDI-TOF analysis. Removal of major serum proteins can be achieved by immobilized dye [15] or immunoaffinity [16,17] and it is a well known approach to improved detection of minor blood proteins in techniques such as two dimensional (2D) electrophoresis [17]. We used the IgY12 microbeads (ProteomLab IgY12, Beckman) on serum samples as recommended by the manufacturer. Briefly, 10 μL of samples were diluted in TBS, added to IgY-microbead spin column and incubated 15 min. The unbound fractions were collected following the centrifugation of the columns. The columns were then rinsed extensively before the elution using a stripping buffer (0.1 M Glycine, pH 2.5) of the bound fractions that were subsequently equilibrated for their pH. The collected fractions were concentrated down to 40 μl on PES ultrafiltration columns with a 10 kDa cut off for hydragel separation and 2D electrophoresis and with a 3 kDa cut off for SELDI-TOF analysis. IgY12 columns are designed to retain by immunocapture 90% to 99.5% of the following 12 serum proteins: Albumin, Transferrin, IgG, Haptoglobin, α1-antitrypsin, a2-macroglobulin, IgA, IgM, Orosomucoid, ApoA-I, ApoA-II and Fibrinogen [18]. Protein quantification of the bound and unbound fractions confirmed that, as expected, 84.3% (sd 2.3%) of the total protein content was retained on the column [17]. This implies that the proteins remaining after depletion have been arithmetically purified by a factor close to 6.4. While 1 μL of undepleted serum was analysed on each SELDI-TOF spot, the immunodepletion was performed on 10 μL of serum and 1/3 of the resulting depleted fraction was analysed, resulting therefore in a 3.3 fold increase in the serum equivalent amount analysed. However, as the depleted fraction after PES concentration had a volume close to 40 μL, the concentration of protein remaining in this fraction is finally divided by 4. We confirmed the quality of the immunocapture by electrophoresis on agarose (Hydragel, Sebia) as illustrated in the Figure 1a. The unbound protein profile obtained, was dramatically modified when compared that of the initial and the bound fractions, which were alike. The different protein fractions were also analyzed using 2D electrophoresis (Figure 1, b–d) using pH 4–7 IPG strips (Amersham) for the first dimension and 4–12% NuPAGE gels (Invitrogen) for the second. Gels were stained with a modified silver nitrate procedure as in Shevchenko et al [19]. The spot/protein pattern was mainly modified in the unbound fraction and we could confirm results obtained by Huang et al [18] who concluded that immunocapture increases the sensitivity of 2D electrophoresis for the detection of low abundant proteins.

Initial, bound and unbound serum fractions were then denatured using a Urea/CHAPS buffer and analysed by SELDI-TOF using CM10 (weak cation exchange) ProteinChip arrays at pH4. To assess the repeatability and reproducibility of the method, the same serum sample was analysed 8 times the same day, and for four consecutive days, respectively. The coefficients of variation (CV) were calculated for the different protein peaks detected on the spectra, as recommended by the Ciphergen company (Table 1). The general repeatability of the SELDI-TOF analysis was in our hand close to 15% which is satisfactory for a manual technique. The reproducibility was lower, with a CV close to 22%, as expected from the additional bias linked to experiments realized on different days. When the immunocapture was realized beforehand, we observed repeatability and reproducibility with CV of 16.7% and 25.3% respectively (Table 1). These values were very close to those obtained without fractionation confirming that this depletion technique was very robust and did not significant increase variability.

**Table 1:** Repeatability and reproducibility of SELDI-TOF spectra before and after immunocapture.

	Minimum CV value	Maximum CV value	∑(CV)2/p MathType@MTEF@5@5@+=feaafiart1ev1aaatCvAUfKttLearuWrP9MDH5MBPbIqV92AaeXatLxBI9gBaebbnrfifHhDYfgasaacH8akY=wiFfYdH8Gipec8Eeeu0xXdbba9frFj0=OqFfea0dXdd9vqai=hGuQ8kuc9pgc9s8qqaq=dirpe0xb9q8qiLsFr0=vr0=vr0dc8meaabaqaciaacaGaaeqabaqabeGadaaakeaadaGcaaqaamaaqaeabaGaeiikaGIaee4qamKaeeOvayLaeiykaKYaaWbaaSqabeaacqaIYaGmaaGccqGGVaWlcqqGWbaCaSqabeqaniabggHiLdaaleqaaaaa@3643@
SELDI-TOF repeatability (p = 33)	9.7	25.9	15.7
SELDI-TOF reproducibility (p = 33)	8.7	52.1	22.2
Y12/SELDI-TOF repeatability (p = 31)	6.4	32.5	16.7
Y12/SELDI-TOF reproducibility (p = 30)	7.8	57.9	25.3

**Legend: **The coefficient of variation (CV) was calculated for the "p" peaks detected in the SELDI-TOF spectra using the ProteinChip Software (Ciphergen Biosystems). The minimum and maximum CV values are indicated on the table, as well as the mean CV values. For the SELDI-TOF and Y12/SELDI-TOF repeatability, a serum sample was analysed 8 times the same day; for the reproducibility, the same sera was analysed for 4 consecutive days.

**Figure 2:**
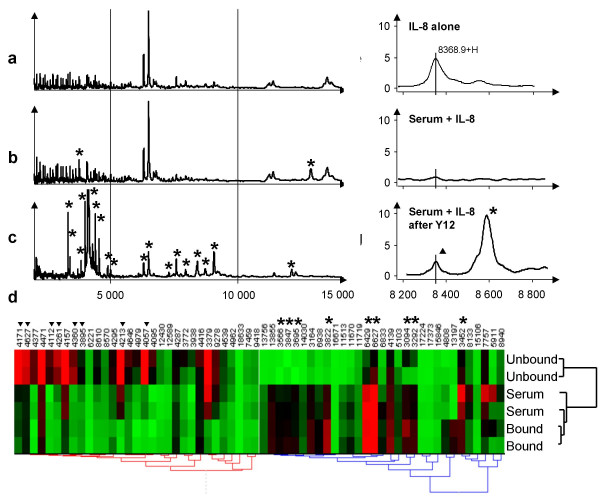
**SELDI-TOF analysis before and after fractionation using IgY12 spin columns**. SELDI-TOF spectrum (between 3,000 and 15,000 m/z) of the original unfractionated serum (panel a), of the unbound (depleted) (panel b) and Y12 bound (panel c) protein fractions. Stars indicated peaks present only in one of the fraction. Panel d: hierarchical clustering using the peaks detected in the a to c panel (in duplicates) using the ProteinChip Software (Ciphergen Biosystems). Numbers on the top represent the m/z values of the peaks. Colors are related to relative distribution between the fractions; green: low level, red: high level, black average level. Triangles and stars indicated peaks that appeared specific of the unbound fraction or present in both bound and original fractions, respectively. Panel e: SEDLI-TOF spectra of pure IL-8 revealing a single peak with an apparent m/z value of 8560. Serum spiked with 0.1 ng/μL of IL-8 was analysed before (panel f) or after (panel g) immunodepletion. A peak (triangle) aligned to that of the IL-8 was only readily detected after depletion in the unbound fraction. The additional peak in this fraction (triangle) corresponded, as in panel c, to an additional serum peak detected only after depletion.

As illustrated Table 2 and Figure 2 for m/z ratio ranging from 3,000 to 15,000, few peaks were present only in the bound fraction (Figure 2b, stars), while the unbound fraction revealed many new peaks (Figure 2c, stars). This comparison of the profiles obtained before and after immunocapture was performed with the Ciphergen Biomarker Wizard software that aligns spectra and identifies differential peaks. However, because of the low mass accuracy of the PBS-II spectrometer when a new peak is close in m/z from a pre-existing peak, we cannot be entirely confident that this peak is new. Nevertheless, the profiles were clearly different in many sections and they were new detected peaks where, before immunodepletion, only the background was detected (Figure 2c and 2g). Pearson factor was also used to asses the general reproducibly of the different profiles and it was above 0.9 (Table 2) confirming the Table 1 results. Hierarchical Cluster analysis (Using Hierarchical Clustering Explorer software, HCE v3 [20]) of the SELDI-TOF data (Figure 2d), revealed the presence of two main clusters, one being composed by serum and bound protein profiles and the second by unbound profiles. This clearly illustrates the fact that some new protein peaks were retained by the column (Figure 2d, stars), while many were detected only in the unbound fraction (Figure 2d, triangles).

**Table 2:** Summary of peak detection in the different fractions

	Detected peaks	Pearson Factor Between each replicate	Number of unique peaks
Serum	32 ± 1	0.90	0
Bound compartment	29 ± 1	0.97	3
Unbound compartment	41 ± 0	0.95	24

**Legend: **Fractionation of serum samples (n = 4) by IgY12 spin columns and SELDI-TOF analysis. Peaks were detected using ProteinChip Software (Ciphergen Biosystems) between 3,000 to 15,000 m/z with a ratio signal/noise of 3. Pearson factor is calculated between replicates of each fraction. Note: unique peaks are those not present in any of the two other fractions.

Using CM10 ProteinChip, purified recombinant IL-8 could be detected as a 8,600 m/z peak (Figure 2e). We used this molecule spiked into the serum to illustrate the potency of the immunodepletion approach for the detection of low abundant proteins. Using a serum supplemented with 0.1 ng/μl of this recombinant IL-8 (11,4 pM), no obvious peak aligned with that of IL-8 was detectable when this it was directly analyzed by SELDI-TOF (Figure 2f). However, after Y12 immunodepletion and SELDI-TOF, a peak likely corresponding to IL-8, which was absent in non spiked serum (not shown), was readily detected in the unbound fraction (Figure 2g, star).

## Conclusion

Removal of the major serum proteins using an immunocapture method allows the SELDI-TOF detection of new peaks, most likely corresponding to low abundant proteins, in the unbound fractions. In the bound fractions, major peaks were still detectable, as well as additional peaks corresponding to proteins co-purified with the 12 proteins retained by the columns. Taken together, our data showed that an approach combining immunocapture of major serum proteins followed by SELDI-TOF is reproducible, versatile, can be applied to a large number of samples and we believe presents a major interest for blood proteome analysis, profiling and biomarker discovery.

## Methods

### Serum samples

Anonymized serum samples that had a normal pattern on Hydragel (see below) were used for this study. Blood was initially collected in Vacutainer tubes without additive, let clot 30 minutes at room temperature and centrifuged for 30 min at 3000 × g. Serum was recovered and frozen at minus 80°C until used.

### IgY12 fractionation

Fractionation of serum proteins was performed as recommended by the manufacturer (Beckmann, ref A24331). Briefly, 10 μL of serum was mixed with 490 μL of Tris buffer solution at pH 7.5 (TBS), added to IgY-microbead spin column and incubated at room temperature for 15 min with rotation. The unbound proteins were collected in a 2 mL eppendorf tube by centrifugation at 400 × g for 30 s. After 3 washes with TBS, bound proteins were eluted in two steps with 500 μL of stripping buffer (0.1 M Glycine, pH 2.5) and the fraction was then neutralised using 100 μL of 0.1 M TrisHCl pH 8. Concentration of the fraction to 40 μL was then realized by PES ultrafiltration (see below). Protein quantitation of the fractions demonstrated that 86% (± 6.5%) of the initial proteins was recovered with a distribution 84,3% (± 2.3%)/15.7% (± 2.3%) of bound/unbound proteins.

### Hydragel analysis

For Hydragel separation and 2D electrophoresis, the unbound and bound proteins were concentrated at 4°C on PES ultrafiltration columns with a 10 kDa cutoff (VIVASPIN 500, Vivascience ref VS0101) in 50 mM Tris pH 8.8. The Hydragel technology from Sebia (ref: 4115) allows the separation and the identification of major serum proteins for clinical application. 5 μL of serum were separated by electrophoresis on agarose following the kit procedure. The proteins were stained by acidic coomassie and the major protein identify by the banding pattern.

### 2D electrophoresis

After the spin columns, samples were mixed with in 200 μL of solubilizing buffer (8 M urea, 1 M thiourea, 4.8% CHAPS, 50 mM DTT). Total protein quantitation was performed using PlusOne 2-D Quant Kit (Amersham Biosciences, ref 80-6483-56). For the first dimension, 20 μg of the samples were diluted in 125 μL of rehydratation buffer (9.8 M urea 4% CHAPS 50 mM DTT and 0.5% IPG buffer 4–7). 7 cm IPG strips (Amersham ref 17-6001-10), covering a pH range of 4 – 7 were rehydrated with this solution during 18 h under low viscosity paraffin oil. For focalisation, the following voltage/time profile was used on a IPG Phor II: 300 V for 2 h, a gradient until 1000 V for 1 h, a gradient until 5000 V for 1 h 30 and 5000 V during 3 h 30. A total of 23250 vh was achieved. For the second dimension, strips were equilibrated for 30 min in 6 M urea, 30% glycerol, 2% SDS, 50 mM Tris pH 8.8,1%DTTand then incubated for an additional 30 min in the same solution except that DTT was replaced by 5% iodoacetamide. After equilibration, proteins were separated in the second dimension in 4–12% NuPAGE gels (Invitrogen). Gels were stained with a modified silver nitrate procedure as in Shevchenko et al [19]. Gels were scanned at 300 dots per inch using Labscan 3 software after a procedure of calibration using kaleidoscope LaserSoft Imaging (Kodak, ref: R020123).

### SELDI-TOF analysis

For SELDI-TOF analysis, the total unbound and bound proteins obtained from 10 μl of serum were concentrated to 40 μl by a 45 min centrifugation at 4°C on PES ultrafiltration columns with a 3 KDa cutoff (Millipore, ref 42403). 1 μl of the initial serum, 12 and 4 μl out of the 40 μl unbound and bound fractions were used for analysis. These volumes have been chosen experimentally after optimization. These samples were diluted 1.5 time with a solution of 8 M Urea, 1% CHAPS and shaken 15 min at room temperature. Denaturated samples were then mixed with 100 μL of binding buffer (100 mM Ammonium Acetate pH4, 0.1% Triton) for application on CM10 (weak cation exchange) ProteinChip (Ciphergen, Fremont, CA). CM10 ProteinChips arrays were pre-equilibrated with 150 μL of binding buffer using in 96 wells bioprocessor and incubated 5 min with gentle agitation. After removing binding buffers from the wells, denaturated samples were added and incubated for 1 h on a plate shaker at room temperature. The wells were washed two times with the binding buffer and one time with 100 mM Ammonium Acetate pH4 during 5 min, followed by a final brief rinse with water. ProteinChip arrays were removed from the bioprocessor and air-dried. Finally, 0.8 μL of saturated sinapinic acid solution was applied twice to each spot and the chips were allowed to air-dry again.

Mass spectrometric analysis was performed by SELDI-TOF in a PBS-II ProteinChip reader (Ciphergen Biosystems) using the same settings for all the samples and for data collection (calibration, focusing mass, laser intensity and detector sensitivity). Each spectrum was an average of at least 100 laser shots. Externally calibration was done with the All-in-1 Protein Standard II (Ciphergen Biosystems). Spectra analysis was carried out using using the ProteinChip software version 3.2 (Ciphergen Biosystems). The background was subtracted using the default software settings. Peaks with a ratio signal/noise above 3 were identified by the ProteinChip Software. After normalization on Total Ion Current (TIC) and quantification, the data were exported to Hierarchical Clustering Explorer Software (HCE v3 [20]). Clusters were processed using Pearson statistical test.

### IL-8 spiking experiment

Human recombinant IL-8 (Calbiochem, ref 407673) was aliquoted at 10 μg/mL in PBS and stored at -80°C. 2.5 μL (25 ng) of this material was analysed by SELDI-TOF to find the corresponding IL-8 peak. For the spiking experiment, 0.1 ng of IL-8 was added per μl of serum (11.4 pM) which was then analysed directly of after immunodepletion as described above.

## Competing interests

The Y12 columns were provided by the Beckman company free of charge for analytical evaluation.

## Authors' contributions

Stéphane Roche participated to the design of the experiment, performed the 2D analysis and drafted the manuscript.

Laurent Tiers carried out the SELDI-TOF analysis.

Monique Provansal and Marie-Thérèse Piva are responsible for serum collection and hydragel and participated in the design of the study.

Sylvain Lehmann is the head of the laboratory, he coordinated the study and wrote the final version of the manuscript.
